# *N*,*N*′-Dibenzyl-*N*,*N*′-dimethyl-*N*′′-(*p*-tol­yl)phospho­ric triamide

**DOI:** 10.1107/S1600536811046046

**Published:** 2011-11-09

**Authors:** Mehrdad Pourayoubi, Behrouz Elahi, Masood Parvez

**Affiliations:** a Department of Chemistry, Ferdowsi University of Mashhad, Mashhad, Iran; b Department of Chemistry, University of Calgary, 2500 University Drive NW, Calgary, Alberta, Canada T2N 1N4

## Abstract

The asymmetric unit of the title compound, C_23_H_28_N_3_OP, contains two independent mol­ecules with significant conformational differences. For example, the torsion angles N—C—C—C involving the *N*-benzyl moieties are 57.3 (7) and 11.6 (8)° in one mol­ecule and 76.5 (7) and 97.4 (7)° in the other. In each mol­ecule, the P atom exhibits a distorted tetra­hedral conformation [the bond angles at P are in the ranges 104.7 (2)–115.2 (2) and 105.1 (2)–115.1 (2)° in the two molecules], and the phosphoryl group and the N—H group adopt an *anti* orientation with respect to one another. In the crystal, mol­ecules are linked *via* N—H⋯O(P) hydrogen bonds, forming a chain parallel to the *a* axis.

## Related literature

For background to the synthesis of related compounds, see: Toghraee *et al.*, (2011 ▶). For related structures, see: Gholivand & Mahzouni (2011 ▶); Pourayoubi *et al.* (2011 ▶).
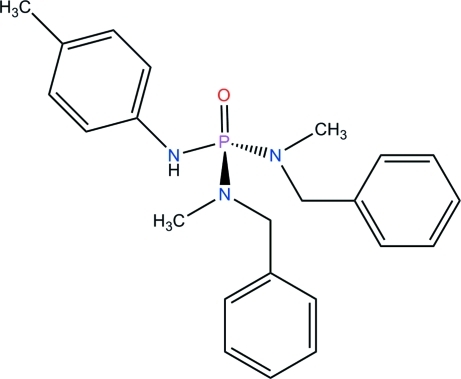


## Experimental

### 

#### Crystal data

C_23_H_28_N_3_OP
*M* *_r_* = 393.45
Monoclinic, 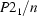

*a* = 9.9621 (3) Å
*b* = 14.8245 (5) Å
*c* = 28.8407 (10) Å
β = 98.2682 (14)°
*V* = 4215.0 (2) Å^3^
*Z* = 8
Mo *K*α radiation
μ = 0.15 mm^−1^
*T* = 173 K
0.06 × 0.05 × 0.04 mm

#### Data collection

Nonius KappaCCD diffractometer with APEXII detector
Absorption correction: multi-scan (*SORTAV*; Blessing, 1997 ▶) *T*_min_ = 0.991, *T*_max_ = 0.994
13508 measured reflections
7788 independent reflections
4450 reflections with *I* > 2σ(*I*)
*R*_int_ = 0.074

#### Refinement

*R*[*F* ^2^ > 2σ(*F* ^2^)] = 0.104
*wR*(*F* ^2^) = 0.199
*S* = 1.15
7788 reflections
508 parameters
H-atom parameters constrained
Δρ_max_ = 0.32 e Å^−3^
Δρ_min_ = −0.33 e Å^−3^

### 

Data collection: *COLLECT* (Hooft, 1998 ▶); cell refinement: *DENZO* (Otwinowski & Minor, 1997 ▶); data reduction: *SCALEPACK* (Otwinowski & Minor, 1997 ▶); program(s) used to solve structure: *SIR92* (Altomare *et al.*, 1993 ▶); program(s) used to refine structure: *SHELXL97* (Sheldrick, 2008 ▶); molecular graphics: *Mercury* (Macrae *et al.*, 2008 ▶); software used to prepare material for publication: *SHELXL97* and *enCIFer* (Allen *et al.*, 2004 ▶).

## Supplementary Material

Crystal structure: contains datablock(s) global, I. DOI: 10.1107/S1600536811046046/nc2247sup1.cif

Structure factors: contains datablock(s) I. DOI: 10.1107/S1600536811046046/nc2247Isup2.hkl

Additional supplementary materials:  crystallographic information; 3D view; checkCIF report

## Figures and Tables

**Table 1:** Hydrogen-bond geometry (Å, °)

*D*—H⋯*A*	*D*—H	H⋯*A*	*D*⋯*A*	*D*—H⋯*A*
N3—H3*N*⋯O2^i^	0.88	2.05	2.846 (6)	149
N6—H6*N*⋯O1^ii^	0.88	2.00	2.799 (6)	151

Symmetry codes: (i) 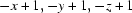; (ii) 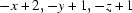.

**Fig. 1.**
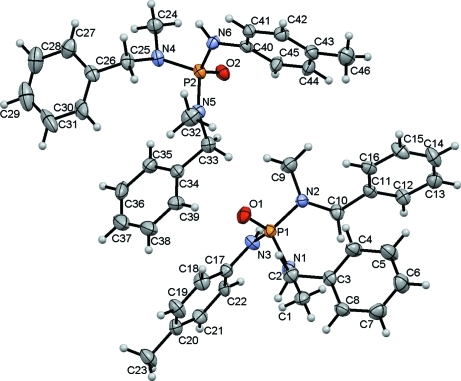
An ORTEP plot of the title molecule with labeling. Displacement ellipsoids are given at 50% probability level.

**Fig. 2.**
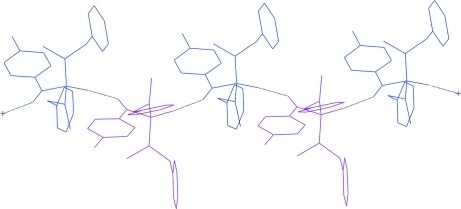
Part of the crystal packing with hydrogen bonds shown as dotted lines. The symmetrically independent molecules are shown as different colors. Only H atoms involved in hydrogen bonds are shown.

## supplementary crystallographic information

### Comment

In continuation of our investigations on the synthesis of new phosphoramidates (Toghraee *et al.*, 2011; Pourayoubi *et al.*, 2011) we now report the synthesis and crystal structure of the title phosphoric triamide.

The asymmetric unit of the title compound (Fig. 1) contains two independent molecules with significant conformational differences. The torsion angles N1–C2–C3–C4 and N2–C10–C11–C16 involving the N-benzyl moiety in one molecule are: 57.3 (7) and 11.6 (8)°, respectively, compared to the corresponding angles in the second molecule with values 76.5 (7) and 97.4 (7)° (for torsion angles N5–C33–C34–C35 and N4–C25–C26–C31, respectively). In each molecule, the P atom adopts a distorted tetrahedral coordination P(═O)(N)(N)_2_ environment with the bond angles in the range of 104.7 (2)° to 115.2 (2)° for P1 and 105.1 (2)° to 115.1 (2)° for P2. The P═O and P—N bond lengths and the P—N—C bond angles are within the expected values for analogous compounds reported in the literature (Gholivand *et al.*, 2011). In both molecules, the P—N bond lengths of the P(O)[NHC_6_H_4_(4-CH_3_)] fragments (1.646 (5) Å and 1.653 (4) Å, respectively, for molecules labeled with P1 and P2) are between the values of the P—N bonds in the P(O)[N(CH_3_)(CH_2_C_6_H_5_)]_2_ moieties (P1: 1.639 (5) Å & 1.660 (5) Å and P2: 1.634 (5) Å & 1.661 (5) Å).

In the crystal packing, the molecules are hydrogen bonded to each other resulting in a chain along to the *a* axis in an alternating sequence (Fig. 2).

### Experimental

To a solution of 4-CH_3_—C_6_H_4_NHP(O)Cl_2_ in chloroform, a solution of *N*-methylbenzylamine in chloroform was added dropwise at 273 K. After stirring for 4 h, the solvent was evaporated at room temperature and the obtained solid was washed with distilled water to remove *N*-methylbenzylamine hydrochloride salt. Single crystals, suitable for X-ray crystallography, were obtained from a solution of title compound in chloroform after slow evaporation at room temperature.

### Refinement

The H-atoms were included at geometrically idealized positions with N—H distances 0.88 Å and C—H distances = 0.95, 0.98 and 0.99 Å for aryl, methyl and methylene type H-atoms, respectively. The methyl H-atoms at C1 were disordered over six sites with equal site occupancy factors. The H-atoms were assigned *U*_iso_ = 1.5 times *U*_eq_ C1 atom and 1.2 times *U*_eq_ of the rest of the parent atoms (C/N). The ADSYMM option in Platon suggests a pseudo-translation in the direction of the c-axis. Indeed the structure can also be refined in the subcell which leads to a strong disorder of the phenyl rings and practically no improvement of the reliability factors. This is in agreement with the results of the structure determination of the supercell, which shows that the torsion of the phenyl rings is different. Therefore, we decided to perform the refinement in the supercell. In this context it is noted that the intensity of the super structure reflections is very low and therefore, only about 60% of all independent reflections are observed, which might also be responsible for the low reliability factors.

### Crystal data

**Table:**

C_23_H_28_N_3_OP	*F*(000) = 1680
*M**_r_* = 393.45	*D*_x_ = 1.240 Mg m^−^^3^
Monoclinic, *P*2_1_/*n*	Mo *K*α radiation, λ = 0.71073 Å
Hall symbol: -P 2yn	Cell parameters from 6919 reflections
*a* = 9.9621 (3) Å	θ = 1.0–26.0°
*b* = 14.8245 (5) Å	µ = 0.15 mm^−^^1^
*c* = 28.8407 (10) Å	*T* = 173 K
β = 98.2682 (14)°	Prism, colorless
*V* = 4215.0 (2) Å^3^	0.06 × 0.05 × 0.04 mm
*Z* = 8	

### Data collection

**Table:**

Nonius KappaCCD diffractometer with APEXII detector	7788 independent reflections
Radiation source: fine-focus sealed tube	4450 reflections with *I* > 2σ(*I*)
graphite	*R*_int_ = 0.074
ω and φ scans	θ_max_ = 26.0°, θ_min_ = 2.1°
Absorption correction: multi-scan (*SORTAV*; Blessing, 1997)	*h* = −12→12
*T*_min_ = 0.991, *T*_max_ = 0.994	*k* = −18→18
13508 measured reflections	*l* = −35→35

### Refinement

**Table:**

Refinement on *F*^2^	Primary atom site location: structure-invariant direct methods
Least-squares matrix: full	Secondary atom site location: difference Fourier map
*R*[*F*^2^ > 2σ(*F*^2^)] = 0.104	Hydrogen site location: inferred from neighbouring sites
*wR*(*F*^2^) = 0.199	H-atom parameters constrained
*S* = 1.15	*w* = 1/[σ^2^(*F*_o_^2^) + 17.8*P*] where *P* = (*F*_o_^2^ + 2*F*_c_^2^)/3
7788 reflections	(Δ/σ)_max_ = 0.002
508 parameters	Δρ_max_ = 0.32 e Å^−^^3^
0 restraints	Δρ_min_ = −0.33 e Å^−^^3^

### Special details

**Table:**

Experimental. IR (KBr, cm^-1^): 3178, 3014, 2913, 1610, 1451, 1326, 1187, 1005, 942, 817.
Geometry. All e.s.d.'s (except the e.s.d. in the dihedral angle between two l.s. planes) are estimated using the full covariance matrix. The cell e.s.d.'s are taken into account individually in the estimation of e.s.d.'s in distances, angles and torsion angles; correlations between e.s.d.'s in cell parameters are only used when they are defined by crystal symmetry. An approximate (isotropic) treatment of cell e.s.d.'s is used for estimating e.s.d.'s involving l.s. planes.
Refinement. Refinement of *F*^2^ against ALL reflections. The weighted *R*-factor *wR* and goodness of fit *S* are based on *F*^2^, conventional *R*-factors *R* are based on *F*, with *F* set to zero for negative *F*^2^. The threshold expression of *F*^2^ > σ(*F*^2^) is used only for calculating *R*-factors(gt) *etc*. and is not relevant to the choice of reflections for refinement. *R*-factors based on *F*^2^ are statistically about twice as large as those based on *F*, and *R*- factors based on ALL data will be even larger.

### Fractional atomic coordinates and isotropic or equivalent isotropic displacement parameters (Å^2^)

**Table:**

	*x*	*y*	*z*	*U*_iso_*/*U*_eq_	Occ. (<1)
P1	0.79811 (14)	0.81400 (11)	0.52504 (5)	0.0261 (3)	
O1	0.9321 (4)	0.7937 (3)	0.51123 (14)	0.0364 (10)	
N1	0.7853 (4)	0.9218 (3)	0.53627 (16)	0.0298 (11)	
N2	0.7750 (4)	0.7533 (3)	0.57164 (15)	0.0274 (11)	
N3	0.6671 (4)	0.7895 (3)	0.48547 (15)	0.0295 (11)	
H3N	0.6123	0.7464	0.4922	0.035*	
C1	0.6567 (6)	0.9707 (4)	0.5311 (2)	0.0381 (15)	
H1A	0.5820	0.9286	0.5215	0.057*	0.50
H1B	0.6571	1.0177	0.5072	0.057*	0.50
H1C	0.6449	0.9985	0.5610	0.057*	0.50
H1D	0.6740	1.0345	0.5383	0.057*	0.50
H1E	0.5989	0.9455	0.5526	0.057*	0.50
H1F	0.6111	0.9647	0.4988	0.057*	0.50
C2	0.9044 (6)	0.9791 (4)	0.5503 (2)	0.0349 (15)	
H2A	0.9046	1.0287	0.5273	0.042*	
H2B	0.9875	0.9429	0.5495	0.042*	
C3	0.9086 (5)	1.0190 (4)	0.59821 (19)	0.0270 (13)	
C4	0.9063 (6)	0.9621 (4)	0.6366 (2)	0.0386 (15)	
H4	0.9031	0.8985	0.6324	0.046*	
C5	0.9086 (7)	0.9988 (4)	0.6806 (2)	0.0467 (17)	
H5	0.9068	0.9599	0.7067	0.056*	
C6	0.9135 (7)	1.0905 (5)	0.6874 (2)	0.0464 (17)	
H6	0.9155	1.1148	0.7180	0.056*	
C7	0.9153 (6)	1.1463 (5)	0.6501 (2)	0.0432 (17)	
H7	0.9192	1.2098	0.6546	0.052*	
C8	0.9116 (6)	1.1112 (4)	0.6054 (2)	0.0340 (14)	
H8	0.9111	1.1509	0.5795	0.041*	
C9	0.7891 (6)	0.6548 (4)	0.5673 (2)	0.0397 (16)	
H9A	0.8097	0.6280	0.5985	0.048*	
H9B	0.8628	0.6415	0.5492	0.048*	
H9C	0.7040	0.6295	0.5512	0.048*	
C10	0.6726 (5)	0.7793 (4)	0.60118 (19)	0.0323 (14)	
H10A	0.5884	0.7450	0.5908	0.039*	
H10B	0.6515	0.8441	0.5961	0.039*	
C11	0.7145 (5)	0.7634 (4)	0.65308 (19)	0.0278 (13)	
C12	0.6183 (6)	0.7749 (4)	0.68242 (19)	0.0328 (14)	
H12	0.5273	0.7882	0.6695	0.039*	
C13	0.6524 (6)	0.7673 (4)	0.7307 (2)	0.0388 (15)	
H13	0.5852	0.7760	0.7505	0.047*	
C14	0.7856 (6)	0.7468 (5)	0.7501 (2)	0.0447 (17)	
H14	0.8099	0.7422	0.7831	0.054*	
C15	0.8803 (6)	0.7335 (4)	0.7208 (2)	0.0399 (16)	
H15	0.9706	0.7180	0.7336	0.048*	
C16	0.8458 (6)	0.7424 (4)	0.67252 (19)	0.0308 (13)	
H16	0.9132	0.7341	0.6527	0.037*	
C17	0.6360 (5)	0.8324 (4)	0.44145 (19)	0.0270 (13)	
C18	0.7352 (6)	0.8716 (4)	0.4181 (2)	0.0368 (15)	
H18	0.8276	0.8695	0.4320	0.044*	
C19	0.7010 (6)	0.9127 (5)	0.3758 (2)	0.0420 (17)	
H19	0.7706	0.9383	0.3606	0.050*	
C20	0.5668 (6)	0.9183 (4)	0.3542 (2)	0.0355 (15)	
C21	0.4688 (6)	0.8791 (4)	0.3772 (2)	0.0360 (15)	
H21	0.3763	0.8818	0.3634	0.043*	
C22	0.5024 (5)	0.8364 (4)	0.41954 (19)	0.0277 (13)	
H22	0.4330	0.8091	0.4341	0.033*	
C23	0.5297 (7)	0.9651 (5)	0.3071 (2)	0.0492 (18)	
H23A	0.4310	0.9646	0.2982	0.059*	
H23B	0.5723	0.9333	0.2832	0.059*	
H23C	0.5621	1.0276	0.3094	0.059*	
P2	0.70017 (14)	0.31166 (11)	0.47176 (5)	0.0258 (3)	
O2	0.5670 (4)	0.2915 (3)	0.48617 (13)	0.0315 (10)	
N4	0.7215 (4)	0.2499 (3)	0.42532 (15)	0.0286 (11)	
N5	0.7131 (4)	0.4189 (3)	0.45992 (16)	0.0286 (11)	
N6	0.8327 (4)	0.2883 (3)	0.51137 (15)	0.0277 (11)	
H6N	0.8884	0.2455	0.5050	0.033*	
C24	0.6886 (6)	0.1534 (4)	0.4276 (2)	0.0378 (15)	
H24A	0.6478	0.1324	0.3966	0.045*	
H24B	0.6244	0.1444	0.4500	0.045*	
H24C	0.7717	0.1192	0.4379	0.045*	
C25	0.8327 (5)	0.2691 (4)	0.39825 (19)	0.0323 (14)	
H25A	0.9089	0.2275	0.4085	0.039*	
H25B	0.8654	0.3314	0.4052	0.039*	
C26	0.7919 (5)	0.2594 (5)	0.3457 (2)	0.0330 (14)	
C27	0.7932 (6)	0.1764 (5)	0.3239 (2)	0.0428 (16)	
H27	0.8191	0.1239	0.3418	0.051*	
C28	0.7561 (7)	0.1696 (6)	0.2749 (2)	0.058 (2)	
H28	0.7560	0.1125	0.2600	0.070*	
C29	0.7202 (7)	0.2455 (8)	0.2489 (3)	0.067 (3)	
H29	0.6954	0.2412	0.2160	0.080*	
C30	0.7202 (7)	0.3267 (7)	0.2705 (2)	0.064 (2)	
H30	0.6956	0.3792	0.2524	0.076*	
C31	0.7554 (6)	0.3344 (5)	0.3183 (2)	0.0442 (17)	
H31	0.7545	0.3920	0.3327	0.053*	
C32	0.8413 (6)	0.4676 (4)	0.4625 (2)	0.0408 (16)	
H32A	0.9169	0.4250	0.4691	0.061*	0.50
H32B	0.8471	0.5126	0.4876	0.061*	0.50
H32C	0.8461	0.4978	0.4326	0.061*	0.50
H32D	0.8232	0.5320	0.4571	0.061*	0.50
H32E	0.8929	0.4443	0.4386	0.061*	0.50
H32F	0.8940	0.4591	0.4936	0.061*	0.50
C33	0.5917 (6)	0.4758 (4)	0.4501 (2)	0.0359 (15)	
H33A	0.5993	0.5263	0.4728	0.043*	
H33B	0.5111	0.4397	0.4548	0.043*	
C34	0.5701 (6)	0.5140 (4)	0.4010 (2)	0.0313 (14)	
C35	0.5201 (6)	0.4618 (4)	0.3632 (2)	0.0389 (15)	
H35	0.5000	0.4001	0.3677	0.047*	
C36	0.4983 (7)	0.4981 (5)	0.3182 (2)	0.0494 (19)	
H36	0.4652	0.4609	0.2923	0.059*	
C37	0.5248 (7)	0.5884 (5)	0.3113 (3)	0.055 (2)	
H37	0.5090	0.6137	0.2807	0.066*	
C38	0.5743 (7)	0.6414 (5)	0.3492 (2)	0.0494 (18)	
H38	0.5931	0.7034	0.3450	0.059*	
C39	0.5964 (6)	0.6037 (4)	0.3934 (2)	0.0369 (15)	
H39	0.6307	0.6406	0.4193	0.044*	
C40	0.8616 (5)	0.3334 (4)	0.55505 (18)	0.0261 (13)	
C41	0.9962 (5)	0.3371 (4)	0.57746 (19)	0.0268 (13)	
H41	1.0668	0.3104	0.5632	0.032*	
C42	1.0257 (6)	0.3798 (4)	0.6203 (2)	0.0322 (14)	
H42	1.1172	0.3813	0.6351	0.039*	
C43	0.9261 (6)	0.4207 (4)	0.6426 (2)	0.0350 (15)	
C44	0.7931 (6)	0.4161 (4)	0.6199 (2)	0.0389 (16)	
H44	0.7226	0.4436	0.6339	0.047*	
C45	0.7613 (6)	0.3722 (4)	0.5770 (2)	0.0335 (14)	
H45	0.6694	0.3688	0.5627	0.040*	
C46	0.9598 (7)	0.4691 (5)	0.6886 (2)	0.0507 (19)	
H46A	0.8840	0.4632	0.7066	0.061*	
H46B	0.9759	0.5331	0.6828	0.061*	
H46C	1.0416	0.4426	0.7063	0.061*	

### Atomic displacement parameters (Å^2^)

**Table:**

	*U*^11^	*U*^22^	*U*^33^	*U*^12^	*U*^13^	*U*^23^
P1	0.0268 (8)	0.0310 (9)	0.0199 (7)	0.0050 (7)	0.0011 (6)	−0.0013 (6)
O1	0.027 (2)	0.050 (3)	0.032 (2)	0.011 (2)	0.0028 (17)	−0.007 (2)
N1	0.025 (2)	0.035 (3)	0.028 (3)	0.009 (2)	0.001 (2)	−0.006 (2)
N2	0.035 (3)	0.025 (3)	0.021 (2)	0.003 (2)	−0.001 (2)	−0.002 (2)
N3	0.030 (3)	0.032 (3)	0.025 (3)	−0.003 (2)	0.001 (2)	0.005 (2)
C1	0.041 (4)	0.039 (4)	0.034 (3)	0.016 (3)	0.003 (3)	0.000 (3)
C2	0.032 (3)	0.035 (4)	0.038 (4)	0.000 (3)	0.007 (3)	−0.001 (3)
C3	0.020 (3)	0.033 (3)	0.029 (3)	−0.002 (2)	0.006 (2)	0.002 (3)
C4	0.053 (4)	0.027 (3)	0.033 (3)	−0.002 (3)	−0.004 (3)	−0.001 (3)
C5	0.069 (5)	0.035 (4)	0.032 (4)	−0.010 (3)	−0.004 (3)	0.004 (3)
C6	0.058 (4)	0.048 (4)	0.031 (4)	−0.008 (4)	0.000 (3)	−0.006 (3)
C7	0.051 (4)	0.031 (4)	0.046 (4)	0.000 (3)	0.002 (3)	−0.006 (3)
C8	0.037 (3)	0.031 (4)	0.034 (4)	0.002 (3)	0.007 (3)	0.009 (3)
C9	0.047 (4)	0.034 (4)	0.038 (4)	0.006 (3)	0.005 (3)	−0.003 (3)
C10	0.028 (3)	0.040 (4)	0.027 (3)	0.001 (3)	0.002 (2)	−0.001 (3)
C11	0.031 (3)	0.026 (3)	0.026 (3)	−0.004 (3)	0.003 (2)	0.004 (3)
C12	0.031 (3)	0.038 (4)	0.029 (3)	−0.003 (3)	0.004 (2)	0.003 (3)
C13	0.044 (4)	0.036 (4)	0.039 (4)	0.000 (3)	0.012 (3)	−0.001 (3)
C14	0.054 (4)	0.056 (4)	0.023 (3)	−0.010 (4)	0.003 (3)	0.006 (3)
C15	0.033 (3)	0.050 (4)	0.033 (3)	−0.006 (3)	−0.007 (3)	0.011 (3)
C16	0.033 (3)	0.026 (3)	0.033 (3)	−0.003 (3)	0.003 (2)	0.005 (3)
C17	0.026 (3)	0.029 (3)	0.025 (3)	0.002 (3)	0.001 (2)	−0.003 (3)
C18	0.025 (3)	0.048 (4)	0.037 (4)	−0.003 (3)	0.003 (3)	0.000 (3)
C19	0.046 (4)	0.049 (4)	0.031 (3)	−0.013 (3)	0.003 (3)	0.009 (3)
C20	0.043 (4)	0.039 (4)	0.023 (3)	0.003 (3)	0.003 (3)	−0.002 (3)
C21	0.036 (3)	0.041 (4)	0.027 (3)	0.009 (3)	−0.007 (3)	−0.003 (3)
C22	0.026 (3)	0.034 (3)	0.025 (3)	−0.002 (3)	0.010 (2)	0.001 (3)
C23	0.064 (5)	0.054 (5)	0.028 (4)	−0.005 (4)	0.001 (3)	0.012 (3)
P2	0.0239 (7)	0.0327 (9)	0.0204 (7)	−0.0018 (7)	0.0021 (6)	0.0029 (7)
O2	0.026 (2)	0.041 (3)	0.027 (2)	−0.0002 (18)	0.0027 (16)	0.0055 (19)
N4	0.029 (3)	0.036 (3)	0.021 (2)	−0.007 (2)	0.005 (2)	−0.002 (2)
N5	0.029 (3)	0.031 (3)	0.026 (3)	0.000 (2)	0.004 (2)	0.004 (2)
N6	0.028 (3)	0.029 (3)	0.025 (2)	0.004 (2)	0.0017 (19)	−0.006 (2)
C24	0.042 (4)	0.036 (4)	0.036 (4)	−0.005 (3)	0.007 (3)	−0.001 (3)
C25	0.025 (3)	0.046 (4)	0.026 (3)	−0.001 (3)	0.001 (2)	−0.003 (3)
C26	0.022 (3)	0.050 (4)	0.027 (3)	0.001 (3)	0.002 (2)	0.006 (3)
C27	0.037 (3)	0.047 (4)	0.047 (4)	−0.006 (3)	0.015 (3)	−0.010 (3)
C28	0.050 (4)	0.084 (6)	0.043 (4)	−0.020 (4)	0.016 (3)	−0.023 (4)
C29	0.047 (5)	0.122 (8)	0.032 (4)	−0.015 (5)	0.006 (3)	−0.011 (5)
C30	0.042 (4)	0.118 (8)	0.032 (4)	0.011 (5)	0.010 (3)	0.030 (5)
C31	0.031 (3)	0.068 (5)	0.039 (4)	0.005 (3)	0.022 (3)	0.008 (3)
C32	0.033 (3)	0.039 (4)	0.051 (4)	−0.008 (3)	0.005 (3)	−0.004 (3)
C33	0.039 (4)	0.037 (4)	0.032 (3)	0.002 (3)	0.009 (3)	0.001 (3)
C34	0.032 (3)	0.034 (4)	0.029 (3)	0.007 (3)	0.007 (3)	0.003 (3)
C35	0.048 (4)	0.035 (4)	0.032 (3)	0.009 (3)	0.001 (3)	−0.003 (3)
C36	0.058 (4)	0.064 (5)	0.025 (3)	0.026 (4)	0.002 (3)	−0.011 (3)
C37	0.060 (5)	0.065 (5)	0.041 (4)	0.026 (4)	0.016 (3)	0.018 (4)
C38	0.056 (4)	0.049 (5)	0.045 (4)	0.017 (4)	0.013 (3)	0.018 (4)
C39	0.037 (4)	0.035 (4)	0.037 (4)	0.007 (3)	0.004 (3)	0.005 (3)
C40	0.034 (3)	0.023 (3)	0.019 (3)	0.001 (2)	−0.003 (2)	0.001 (2)
C41	0.027 (3)	0.029 (3)	0.025 (3)	0.002 (3)	0.006 (2)	0.003 (3)
C42	0.032 (3)	0.032 (4)	0.031 (3)	0.001 (3)	−0.001 (3)	−0.001 (3)
C43	0.046 (4)	0.033 (4)	0.025 (3)	0.003 (3)	−0.001 (3)	0.001 (3)
C44	0.045 (4)	0.040 (4)	0.032 (3)	0.011 (3)	0.009 (3)	−0.009 (3)
C45	0.031 (3)	0.040 (4)	0.029 (3)	0.011 (3)	0.005 (3)	−0.006 (3)
C46	0.067 (5)	0.044 (4)	0.039 (4)	−0.005 (4)	0.001 (3)	−0.008 (3)

### Geometric parameters (Å, °)

**Table:**

P1—O1	1.478 (4)	P2—O2	1.476 (4)
P1—N1	1.639 (5)	P2—N5	1.634 (5)
P1—N3	1.646 (5)	P2—N6	1.653 (4)
P1—N2	1.660 (5)	P2—N4	1.661 (5)
N1—C1	1.460 (7)	N4—C24	1.472 (7)
N1—C2	1.469 (7)	N4—C25	1.472 (7)
N2—C10	1.472 (7)	N5—C32	1.460 (7)
N2—C9	1.474 (7)	N5—C33	1.469 (7)
N3—C17	1.414 (7)	N6—C40	1.418 (7)
N3—H3N	0.8800	N6—H6N	0.8800
C1—H1A	0.9800	C24—H24A	0.9800
C1—H1B	0.9800	C24—H24B	0.9800
C1—H1C	0.9800	C24—H24C	0.9800
C1—H1D	0.9800	C25—C26	1.518 (7)
C1—H1E	0.9800	C25—H25A	0.9900
C1—H1F	0.9800	C25—H25B	0.9900
C2—C3	1.497 (8)	C26—C31	1.382 (9)
C2—H2A	0.9900	C26—C27	1.383 (9)
C2—H2B	0.9900	C27—C28	1.410 (9)
C3—C8	1.383 (8)	C27—H27	0.9500
C3—C4	1.394 (8)	C28—C29	1.371 (11)
C4—C5	1.379 (8)	C28—H28	0.9500
C4—H4	0.9500	C29—C30	1.354 (12)
C5—C6	1.374 (9)	C29—H29	0.9500
C5—H5	0.9500	C30—C31	1.379 (9)
C6—C7	1.360 (9)	C30—H30	0.9500
C6—H6	0.9500	C31—H31	0.9500
C7—C8	1.385 (9)	C32—H32A	0.9800
C7—H7	0.9500	C32—H32B	0.9800
C8—H8	0.9500	C32—H32C	0.9800
C9—H9A	0.9800	C32—H32D	0.9800
C9—H9B	0.9800	C32—H32E	0.9800
C9—H9C	0.9800	C32—H32F	0.9800
C10—C11	1.513 (7)	C33—C34	1.510 (8)
C10—H10A	0.9900	C33—H33A	0.9900
C10—H10B	0.9900	C33—H33B	0.9900
C11—C12	1.378 (7)	C34—C35	1.373 (8)
C11—C16	1.382 (7)	C34—C39	1.379 (8)
C12—C13	1.389 (8)	C35—C36	1.392 (8)
C12—H12	0.9500	C35—H35	0.9500
C13—C14	1.397 (8)	C36—C37	1.385 (10)
C13—H13	0.9500	C36—H36	0.9500
C14—C15	1.368 (8)	C37—C38	1.379 (10)
C14—H14	0.9500	C37—H37	0.9500
C15—C16	1.391 (7)	C38—C39	1.379 (8)
C15—H15	0.9500	C38—H38	0.9500
C16—H16	0.9500	C39—H39	0.9500
C17—C22	1.390 (7)	C40—C45	1.384 (8)
C17—C18	1.399 (8)	C40—C41	1.403 (7)
C18—C19	1.364 (8)	C41—C42	1.381 (8)
C18—H18	0.9500	C41—H41	0.9500
C19—C20	1.394 (8)	C42—C43	1.397 (8)
C19—H19	0.9500	C42—H42	0.9500
C20—C21	1.386 (8)	C43—C44	1.393 (8)
C20—C23	1.524 (8)	C43—C46	1.502 (8)
C21—C22	1.373 (8)	C44—C45	1.393 (8)
C21—H21	0.9500	C44—H44	0.9500
C22—H22	0.9500	C45—H45	0.9500
C23—H23A	0.9800	C46—H46A	0.9800
C23—H23B	0.9800	C46—H46B	0.9800
C23—H23C	0.9800	C46—H46C	0.9800
			
O1—P1—N1	110.5 (3)	O2—P2—N5	110.9 (2)
O1—P1—N3	115.2 (2)	O2—P2—N6	115.1 (2)
N1—P1—N3	106.0 (2)	N5—P2—N6	105.6 (2)
O1—P1—N2	110.1 (2)	O2—P2—N4	109.9 (2)
N1—P1—N2	110.1 (2)	N5—P2—N4	110.1 (2)
N3—P1—N2	104.7 (2)	N6—P2—N4	105.1 (2)
C1—N1—C2	113.8 (5)	C24—N4—C25	113.7 (5)
C1—N1—P1	123.7 (4)	C24—N4—P2	116.3 (4)
C2—N1—P1	122.4 (4)	C25—N4—P2	121.0 (4)
C10—N2—C9	113.0 (5)	C32—N5—C33	114.6 (5)
C10—N2—P1	121.0 (4)	C32—N5—P2	124.4 (4)
C9—N2—P1	116.2 (4)	C33—N5—P2	120.8 (4)
C17—N3—P1	125.0 (4)	C40—N6—P2	123.7 (4)
C17—N3—H3N	117.5	C40—N6—H6N	118.1
P1—N3—H3N	117.5	P2—N6—H6N	118.1
N1—C1—H1A	109.5	N4—C24—H24A	109.5
N1—C1—H1B	109.5	N4—C24—H24B	109.5
H1A—C1—H1B	109.5	H24A—C24—H24B	109.5
N1—C1—H1C	109.5	N4—C24—H24C	109.5
H1A—C1—H1C	109.5	H24A—C24—H24C	109.5
H1B—C1—H1C	109.5	H24B—C24—H24C	109.5
N1—C1—H1D	109.5	N4—C25—C26	113.4 (4)
H1A—C1—H1D	141.1	N4—C25—H25A	108.9
H1B—C1—H1D	56.3	C26—C25—H25A	108.9
H1C—C1—H1D	56.3	N4—C25—H25B	108.9
N1—C1—H1E	109.5	C26—C25—H25B	108.9
H1A—C1—H1E	56.3	H25A—C25—H25B	107.7
H1B—C1—H1E	141.1	C31—C26—C27	118.3 (6)
H1C—C1—H1E	56.3	C31—C26—C25	120.3 (6)
H1D—C1—H1E	109.5	C27—C26—C25	121.4 (6)
N1—C1—H1F	109.5	C26—C27—C28	120.1 (7)
H1A—C1—H1F	56.3	C26—C27—H27	119.9
H1B—C1—H1F	56.3	C28—C27—H27	119.9
H1C—C1—H1F	141.1	C29—C28—C27	119.9 (7)
H1D—C1—H1F	109.5	C29—C28—H28	120.0
H1E—C1—H1F	109.5	C27—C28—H28	120.0
N1—C2—C3	113.4 (5)	C30—C29—C28	119.7 (7)
N1—C2—H2A	108.9	C30—C29—H29	120.1
C3—C2—H2A	108.9	C28—C29—H29	120.1
N1—C2—H2B	108.9	C29—C30—C31	121.1 (8)
C3—C2—H2B	108.9	C29—C30—H30	119.5
H2A—C2—H2B	107.7	C31—C30—H30	119.5
C8—C3—C4	118.8 (6)	C30—C31—C26	120.9 (7)
C8—C3—C2	121.7 (5)	C30—C31—H31	119.5
C4—C3—C2	119.4 (5)	C26—C31—H31	119.5
C5—C4—C3	119.5 (6)	N5—C32—H32A	109.5
C5—C4—H4	120.3	N5—C32—H32B	109.5
C3—C4—H4	120.3	H32A—C32—H32B	109.5
C6—C5—C4	121.2 (6)	N5—C32—H32C	109.5
C6—C5—H5	119.4	H32A—C32—H32C	109.5
C4—C5—H5	119.4	H32B—C32—H32C	109.5
C7—C6—C5	119.5 (6)	N5—C32—H32D	109.5
C7—C6—H6	120.2	H32A—C32—H32D	141.1
C5—C6—H6	120.2	H32B—C32—H32D	56.3
C6—C7—C8	120.5 (6)	H32C—C32—H32D	56.3
C6—C7—H7	119.8	N5—C32—H32E	109.5
C8—C7—H7	119.8	H32A—C32—H32E	56.3
C3—C8—C7	120.5 (6)	H32B—C32—H32E	141.1
C3—C8—H8	119.8	H32C—C32—H32E	56.3
C7—C8—H8	119.8	H32D—C32—H32E	109.5
N2—C9—H9A	109.5	N5—C32—H32F	109.5
N2—C9—H9B	109.5	H32A—C32—H32F	56.3
H9A—C9—H9B	109.5	H32B—C32—H32F	56.3
N2—C9—H9C	109.5	H32C—C32—H32F	141.1
H9A—C9—H9C	109.5	H32D—C32—H32F	109.5
H9B—C9—H9C	109.5	H32E—C32—H32F	109.5
N2—C10—C11	114.6 (4)	N5—C33—C34	113.5 (5)
N2—C10—H10A	108.6	N5—C33—H33A	108.9
C11—C10—H10A	108.6	C34—C33—H33A	108.9
N2—C10—H10B	108.6	N5—C33—H33B	108.9
C11—C10—H10B	108.6	C34—C33—H33B	108.9
H10A—C10—H10B	107.6	H33A—C33—H33B	107.7
C12—C11—C16	118.7 (5)	C35—C34—C39	118.1 (6)
C12—C11—C10	118.1 (5)	C35—C34—C33	121.3 (6)
C16—C11—C10	123.2 (5)	C39—C34—C33	120.5 (6)
C11—C12—C13	120.9 (5)	C34—C35—C36	120.9 (6)
C11—C12—H12	119.6	C34—C35—H35	119.6
C13—C12—H12	119.6	C36—C35—H35	119.6
C12—C13—C14	120.0 (6)	C37—C36—C35	120.1 (6)
C12—C13—H13	120.0	C37—C36—H36	120.0
C14—C13—H13	120.0	C35—C36—H36	120.0
C15—C14—C13	118.9 (6)	C38—C37—C36	119.3 (7)
C15—C14—H14	120.5	C38—C37—H37	120.3
C13—C14—H14	120.5	C36—C37—H37	120.3
C14—C15—C16	120.7 (6)	C39—C38—C37	119.6 (7)
C14—C15—H15	119.7	C39—C38—H38	120.2
C16—C15—H15	119.7	C37—C38—H38	120.2
C11—C16—C15	120.7 (6)	C38—C39—C34	122.0 (7)
C11—C16—H16	119.6	C38—C39—H39	119.0
C15—C16—H16	119.6	C34—C39—H39	119.0
C22—C17—C18	117.4 (5)	C45—C40—C41	118.6 (5)
C22—C17—N3	120.0 (5)	C45—C40—N6	122.3 (5)
C18—C17—N3	122.7 (5)	C41—C40—N6	119.1 (5)
C19—C18—C17	120.9 (6)	C42—C41—C40	119.7 (5)
C19—C18—H18	119.5	C42—C41—H41	120.1
C17—C18—H18	119.5	C40—C41—H41	120.1
C18—C19—C20	121.8 (6)	C41—C42—C43	122.5 (5)
C18—C19—H19	119.1	C41—C42—H42	118.7
C20—C19—H19	119.1	C43—C42—H42	118.7
C21—C20—C19	117.3 (6)	C44—C43—C42	116.9 (5)
C21—C20—C23	121.4 (6)	C44—C43—C46	121.0 (6)
C19—C20—C23	121.3 (6)	C42—C43—C46	122.1 (6)
C22—C21—C20	121.4 (5)	C45—C44—C43	121.3 (6)
C22—C21—H21	119.3	C45—C44—H44	119.3
C20—C21—H21	119.3	C43—C44—H44	119.3
C21—C22—C17	121.3 (5)	C40—C45—C44	120.9 (5)
C21—C22—H22	119.4	C40—C45—H45	119.5
C17—C22—H22	119.4	C44—C45—H45	119.5
C20—C23—H23A	109.5	C43—C46—H46A	109.5
C20—C23—H23B	109.5	C43—C46—H46B	109.5
H23A—C23—H23B	109.5	H46A—C46—H46B	109.5
C20—C23—H23C	109.5	C43—C46—H46C	109.5
H23A—C23—H23C	109.5	H46A—C46—H46C	109.5
H23B—C23—H23C	109.5	H46B—C46—H46C	109.5
			
O1—P1—N1—C1	152.6 (4)	O2—P2—N4—C24	−47.0 (5)
N3—P1—N1—C1	27.2 (5)	N5—P2—N4—C24	−169.4 (4)
N2—P1—N1—C1	−85.5 (5)	N6—P2—N4—C24	77.3 (4)
O1—P1—N1—C2	−24.2 (5)	O2—P2—N4—C25	168.0 (4)
N3—P1—N1—C2	−149.6 (4)	N5—P2—N4—C25	45.6 (5)
N2—P1—N1—C2	97.7 (5)	N6—P2—N4—C25	−67.7 (5)
O1—P1—N2—C10	160.9 (4)	O2—P2—N5—C32	155.0 (4)
N1—P1—N2—C10	38.8 (5)	N6—P2—N5—C32	29.8 (5)
N3—P1—N2—C10	−74.8 (4)	N4—P2—N5—C32	−83.2 (5)
O1—P1—N2—C9	−55.5 (5)	O2—P2—N5—C33	−18.8 (5)
N1—P1—N2—C9	−177.7 (4)	N6—P2—N5—C33	−144.0 (4)
N3—P1—N2—C9	68.8 (4)	N4—P2—N5—C33	103.0 (4)
O1—P1—N3—C17	−65.8 (5)	O2—P2—N6—C40	−65.4 (5)
N1—P1—N3—C17	56.7 (5)	N5—P2—N6—C40	57.2 (5)
N2—P1—N3—C17	173.2 (4)	N4—P2—N6—C40	173.7 (4)
C1—N1—C2—C3	65.1 (6)	C24—N4—C25—C26	73.7 (6)
P1—N1—C2—C3	−117.8 (5)	P2—N4—C25—C26	−140.4 (5)
N1—C2—C3—C8	−121.2 (6)	N4—C25—C26—C31	97.4 (7)
N1—C2—C3—C4	57.3 (7)	N4—C25—C26—C27	−84.2 (7)
C8—C3—C4—C5	−0.7 (9)	C31—C26—C27—C28	−0.9 (9)
C2—C3—C4—C5	−179.3 (6)	C25—C26—C27—C28	−179.3 (5)
C3—C4—C5—C6	−0.2 (10)	C26—C27—C28—C29	0.7 (10)
C4—C5—C6—C7	0.4 (11)	C27—C28—C29—C30	−0.2 (11)
C5—C6—C7—C8	0.4 (11)	C28—C29—C30—C31	−0.2 (11)
C4—C3—C8—C7	1.4 (9)	C29—C30—C31—C26	0.1 (10)
C2—C3—C8—C7	179.9 (5)	C27—C26—C31—C30	0.5 (9)
C6—C7—C8—C3	−1.3 (10)	C25—C26—C31—C30	178.9 (5)
C9—N2—C10—C11	72.7 (6)	C32—N5—C33—C34	69.1 (7)
P1—N2—C10—C11	−142.7 (4)	P2—N5—C33—C34	−116.5 (5)
N2—C10—C11—C12	−171.8 (5)	N5—C33—C34—C35	76.5 (7)
N2—C10—C11—C16	11.6 (8)	N5—C33—C34—C39	−105.6 (6)
C16—C11—C12—C13	1.1 (9)	C39—C34—C35—C36	0.9 (9)
C10—C11—C12—C13	−175.7 (6)	C33—C34—C35—C36	178.8 (6)
C11—C12—C13—C14	−0.6 (9)	C34—C35—C36—C37	−1.2 (10)
C12—C13—C14—C15	−0.7 (10)	C35—C36—C37—C38	0.8 (10)
C13—C14—C15—C16	1.6 (10)	C36—C37—C38—C39	−0.1 (10)
C12—C11—C16—C15	−0.3 (9)	C37—C38—C39—C34	−0.1 (10)
C10—C11—C16—C15	176.4 (6)	C35—C34—C39—C38	−0.3 (9)
C14—C15—C16—C11	−1.1 (9)	C33—C34—C39—C38	−178.2 (6)
P1—N3—C17—C22	−152.8 (5)	P2—N6—C40—C45	27.2 (8)
P1—N3—C17—C18	27.5 (8)	P2—N6—C40—C41	−154.3 (4)
C22—C17—C18—C19	0.6 (9)	C45—C40—C41—C42	−0.6 (8)
N3—C17—C18—C19	−179.7 (6)	N6—C40—C41—C42	−179.2 (5)
C17—C18—C19—C20	0.6 (10)	C40—C41—C42—C43	−0.5 (9)
C18—C19—C20—C21	−0.9 (10)	C41—C42—C43—C44	0.6 (9)
C18—C19—C20—C23	179.4 (6)	C41—C42—C43—C46	−178.3 (6)
C19—C20—C21—C22	−0.1 (9)	C42—C43—C44—C45	0.4 (9)
C23—C20—C21—C22	179.6 (6)	C46—C43—C44—C45	179.3 (6)
C20—C21—C22—C17	1.3 (9)	C41—C40—C45—C44	1.6 (9)
C18—C17—C22—C21	−1.5 (9)	N6—C40—C45—C44	−179.8 (6)
N3—C17—C22—C21	178.8 (5)	C43—C44—C45—C40	−1.6 (10)

### Hydrogen-bond geometry (Å, °)

**Table:**

*D*—H···*A*	*D*—H	H···*A*	*D*···*A*	*D*—H···*A*
N3—H3N···O2^i^	0.88	2.05	2.846 (6)	149.
N6—H6N···O1^ii^	0.88	2.00	2.799 (6)	151.

Symmetry codes: (i) −*x*+1, −*y*+1, −*z*+1; (ii) −*x*+2, −*y*+1, −*z*+1.

## References

[bb1] Allen, F. H., Johnson, O., Shields, G. P., Smith, B. R. & Towler, M. (2004). *J. Appl. Cryst.* **37**, 335–338.

[bb2] Altomare, A., Cascarano, G., Giacovazzo, C. & Guagliardi, A. (1993). *J. Appl. Cryst.* **26**, 343–350.

[bb3] Blessing, R. H. (1997). *J. Appl. Cryst.* **30**, 421–426.

[bb4] Gholivand, K. & Mahzouni, H. R. (2011). *Acta Cryst.* B**67**, 238–243. 10.1107/S0108768111010470 21586831

[bb5] Hooft, R. (1998). *COLLECT.* Nonius BV, Delft. The Netherlands.

[bb6] Macrae, C. F., Bruno, I. J., Chisholm, J. A., Edgington, P. R., McCabe, P., Pidcock, E., Rodriguez-Monge, L., Taylor, R., van de Streek, J. & Wood, P. A. (2008). *J. Appl. Cryst.* **41**, 466–470.

[bb7] Otwinowski, Z. & Minor, W. (1997). *Methods in Enzymology*, Vol. 276, *Macromolecular Crystallography*, Part A, edited by C. W. Carter Jr & R. M. Sweet, pp. 307–326. New York: Academic Press.

[bb8] Pourayoubi, M., Fadaei, H. & Parvez, M. (2011). *Acta Cryst.* E**67**, o2046. 10.1107/S1600536811027681 PMC3213494 22091073

[bb9] Sheldrick, G. M. (2008). *Acta Cryst.* A**64**, 112–122. 10.1107/S0108767307043930 18156677

[bb10] Toghraee, M., Pourayoubi, M. & Divjakovic, V. (2011). *Polyhedron*, **30**, 1680–1690.

